# Case Report: Post-mortem Histopathological and Molecular Analyses of the Very First Documented COVID-19-Related Death in Europe

**DOI:** 10.3389/fmed.2021.612758

**Published:** 2021-02-19

**Authors:** Milenko Bogdanović, Ivan Skadrić, Tatjana Atanasijević, Oliver Stojković, Vesna Popović, Slobodan Savić, Zoran Mihailović, Bojana Radnić, Tijana Aćimović, Irina Damjanjuk, Sanja Despotović, Aleksandra Barać

**Affiliations:** ^1^Faculty of Medicine, Institute of Forensic Medicine “Milovan Milovanović”, University of Belgrade, Belgrade, Serbia; ^2^Faculty of Medicine, Institute of Human Genetics, University of Belgrade, Belgrade, Serbia; ^3^Faculty of Medicine, Institute of Histology and Embryology, University of Belgrade, Belgrade, Serbia; ^4^Clinic for Infectious and Tropical Diseases, Clinical Centre of Serbia, Belgrade, Serbia; ^5^Faculty of Medicine, University of Belgrade, Belgrade, Serbia

**Keywords:** COVID-19, SARS-CoV-2, vitreous humor, autopsy, Europe (central)

## Abstract

In Europe, the first case of coronavirus disease (COVID-19) and the first COVID-19-related death were reported in France on January 24th and February 15th, 2020, respectively. Officially, the first case of COVID-19 infection in the Republic of Serbia was registered on March 6th. Herein, we presented the first case of retrospective detection of the Severe Acute Respiratory Syndrome Coronavirus 2 (SARS-CoV-2) in the post-mortem-obtained vitreous humor (VH), which took place on February 5th, 2020. This is the first death in Europe proven to be caused by COVID-19 by means of post-mortem histopathological and molecular analyses. Based on this finding, it appears that SARS-CoV-2 has been spreading faster and started spreading much earlier than it had been considered and that COVID-19 was probably the cause of the much-reported pneumonia of unknown origin in January and February 2020.

## Introduction

In December 2019 and January 2020, doctors around the world encountered an unusual situation—a significantly higher number of patients suffering from pneumonia of unknown origin compared to previous years (1, 2). In December 2019, it was obvious that Wuhan and other cities in China had faced a new public health challenge, recognized as coronavirus disease (COVID-19). The causative agent, identified from throat swab samples on January 7th, 2020, was named Severe Acute Respiratory Syndrome Coronavirus 2 (SARS-CoV-2) and quickly became a serious problem worldwide with a high mortality rate and the need for quarantine (3, 4). In Europe, the first case and the first death caused by COVID-19 were reported in France on January 24th and February 15th, 2020, respectively (5). Officially, the first case of COVID-19 in the Republic of Serbia was registered on March 6th (6). Herein, we presented the first case of retrospective detection of SARS-CoV-2 in the post-mortem-obtained vitreous humor (VH), more than a month before the first case was officially registered in the Balkan region (6)^1^. In addition, this is the first death caused by COVID-19 in Europe proven by post-mortem diagnostics.

## Case Description

On February 5th 2020, a 56-year-old man presented to the hospital with fever, cough, and shortness of breath, which started 5 days before. The socio-epidemiological questionnaire showed^1^ that the patient worked in a construction company, was living in a Belgrade suburb, had not traveled abroad for a long time, had no chronic conditions, but was slightly overweight (BMI 25.2 kg/m^2^). On admission, the patient had a high fever (39.2°C) and hypoxia (PaO2 78%) and was hypotensive (blood pressure 100/50 mm/Hg). Chest radiography revealed massive bilateral pneumonia. The biochemical and blood gas analyses revealed the following (reference ranges are shown in parentheses): white blood cell 1.9 × 10^9^/L (4.40–11.50), platelet 127 × 10^9^/L (150–400), D-dimer 3.9 g/L (2–4), C-reactive protein 229 mg/dL (0–7), pO2 25 (80–100), pCO2 33 (35–45), and CHCO_3_ 21 (22–28). The patient was intubated and connected to mechanical ventilation; unfortunately, he died within a few hours after admission to the hospital. The autopsy was performed the next day, according to the standard procedure. Gross autopsy findings revealed heavy, grossly firm, and rubbery lungs with severe bilateral edema. On the cut section, the lungs were dark red without purulent discharge. The hilar lymph nodes were slightly enlarged. The findings in other organs were unremarkable.

Initially, the death was attributed to pneumonia of unknown origin but after additional diagnostic procedures conducted 3 months later, it was proven that death was caused by COVID-19.

### Histopathological Analysis

**Figure 1:**
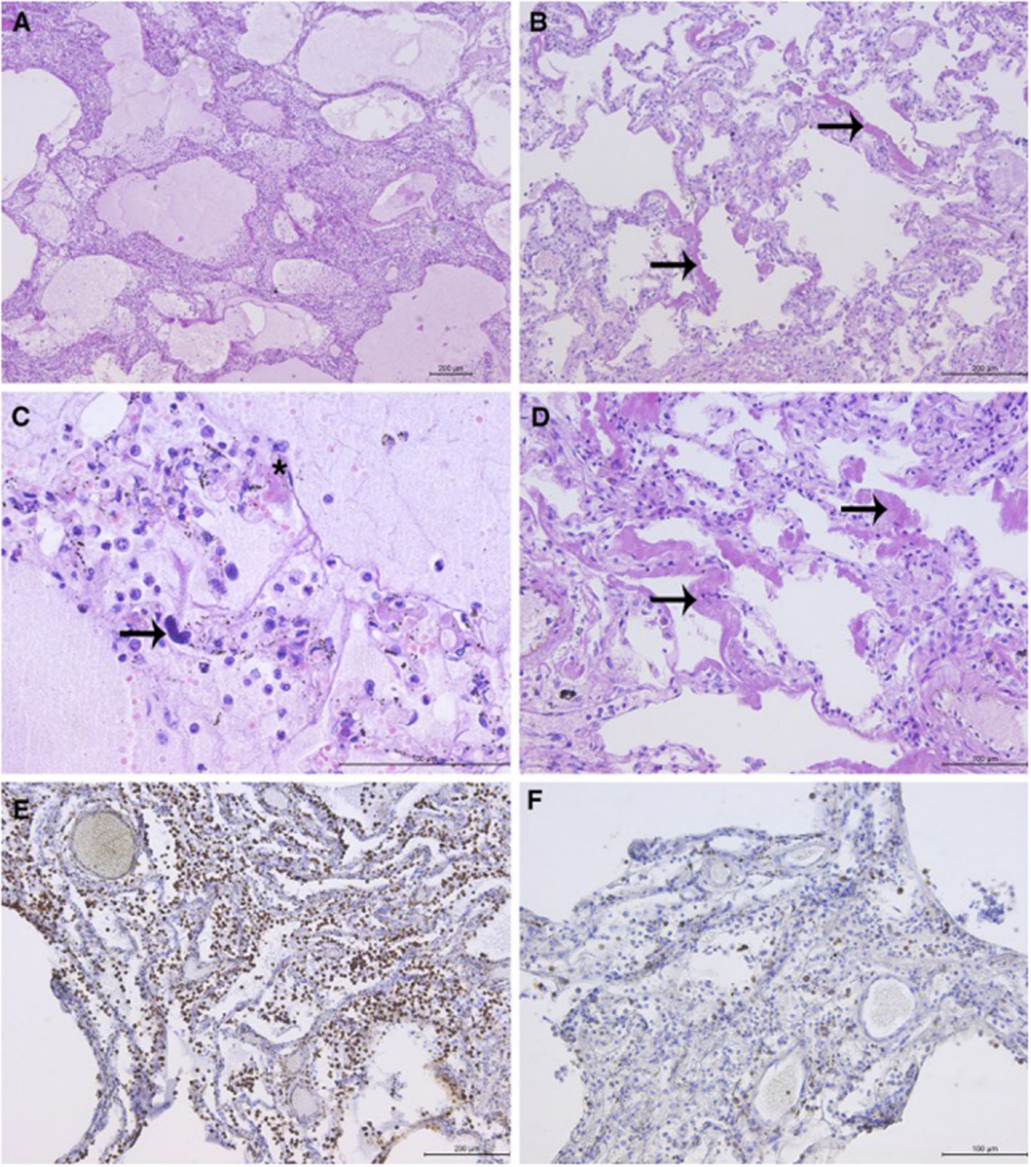
Representative lung sections showing diffuse alveolar damage (DAD): **(A)** DAD with associated pneumonia. Intraalveolar protein-rich edema and inflammatory cells in the alveolar lumen and interstitium. **(B)** Hyaline membranes (arrows) with widening of the alveolar septae. **(C)** Alveolar protein-rich edema. Mixed inflammatory infiltrate with predominance of macrophages. Scattered granulocytes can be observed. Megakaryocyte in the capillary (arrow). Hyaline microthrombus (asterisk). **(D)** Hyaline membranes (arrows), light interstitial fibrosis, and proliferation of type 2 pneumocytes. **(E)** Abundant macrophages in the interstitium and lumen of the alveoli (immunohistochemical staining with CD68 antibody) and **(F)** scattered T-lymphocytes (staining with CD3 antibody). *Microphotographs were taken using the Leica DM4000 B LED light microscope (Leica, Wetzlar, Germany) and Leica DFC295 digital camera (Leica, Heerbrugg, Switzerland).

Histopathological analysis (HP) of the lungs revealed exudative and early organizing phases of diffuse alveolar damage (DAD) in all sections (Figure 1). The findings of intraalveolar protein-rich edema, capillary congestion, and formation of hyaline membranes corresponded to the exudative phase of DAD. DAD was focally associated with fibrinous pneumonia with a mixed inflammatory infiltrate in the lumen of the alveoli and interstitium, consisting of abundant monocytes/macrophages (CD68 staining), rare T-cells (CD3 staining), and scattered granulocytes. Megakaryocytes were observed in the microvasculature. The focal proliferation of type 2 pneumocytes, thickening of the alveolar septae, fibroblast proliferation, and loose interstitial fibrosis corresponded to the early organizing phase of DAD. Few hyaline microthrombi were present in sections. HP findings of other organs were unremarkable (Figure 1).

### Molecular Testing

**Figure 2:**
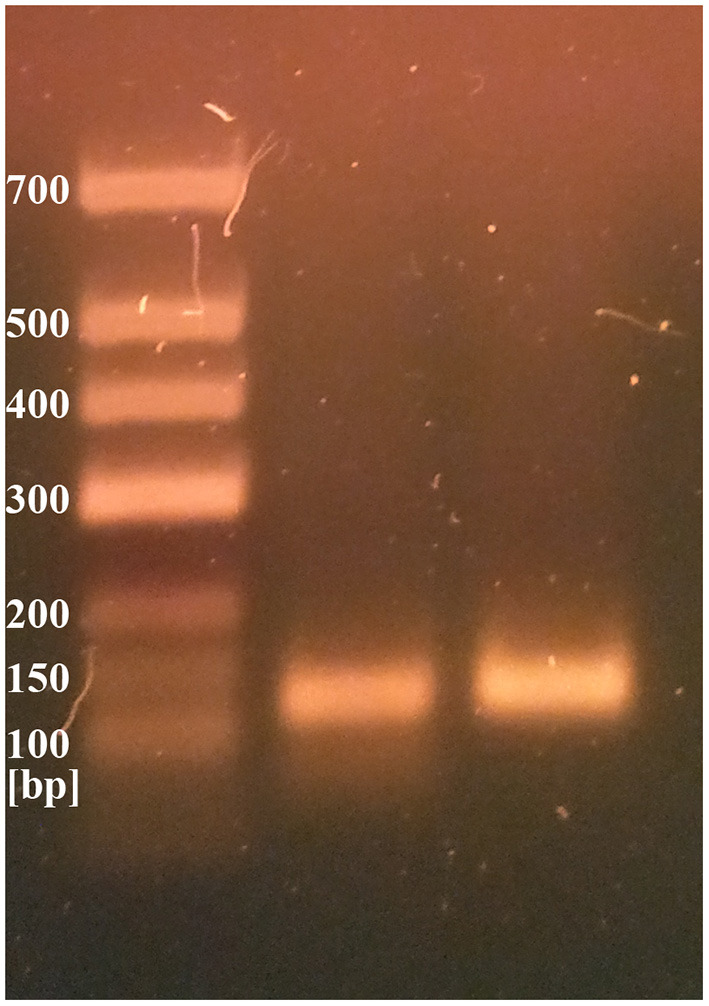
IP2 and IP4 PCR products on 2% agarose gel.

**Figure 3:**
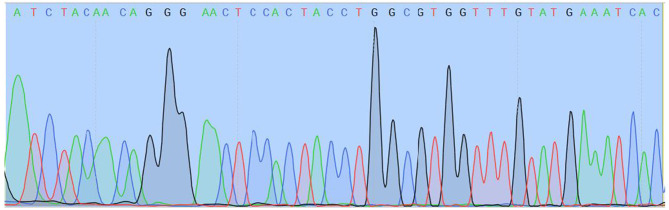
Readable sequence of IP4 amplicon without primer binding sites.

Two different genetic analyses of VH samples (RNA isolation/reverse transcription and Real-Time PCR/amplicon sequencing) were performed with the aim of confirming the validity of results and eliminating the possibility of false-positive results. Two regions of SARS-CoV-2 orf1ab were amplified using the Institute Pasteur primers (IP2 and IP4)^1^. To confirm the specificity, agarose gel extraction of IP4 amplicon and DNA sequencing were performed (Figure 2). The obtained sequence was aligned against reference genomes in the NCBI database using MEGABLAST. IP2 and IP4 regions were amplified with CT values 35 and 34, respectively. PCR products matched the expected lengths for IP2 and IP4 segments, with a minor non-specific band in the P2 lane. MEGABLAST results showed that the sequence without primer binding regions matched SARS-CoV-2 with an E value of 2e−15, as well as 97.92% of percentage identity with NC_045512.2 (Figure 3).

### Confirmatory Assay

Since this was presumably the first confirmed case of COVID-19 in our country, we performed a second molecular analysis of different gene targets of SARS-CoV-2, as suggested by the Center for Disease Control and Prevention. Total RNA was separately isolated from an additional sample of 200 μl of VH. The sequences from the SARS-CoV-2-specific RdRp gene, as well as from the human RNase P gene as internal control, were detected.

## Discussion

The case represents the very first fatal outcome of COVID-19 in Europe and the first post-mortem-confirmed COVID-19 case after retrospective VH analysis. The patient died on February 5th, after 5 days of intensive respiratory symptoms, indicating that he was probably infected in January. The fact that the patient had not traveled abroad and the fact that there were no known epidemiological links with other countries with an active COVID-19 epidemic at that moment indicate that SARS-CoV-2 had probably already been spreading among the population in the Balkans at the beginning of 2020. The study of Deslandes et al. (7) confirmed the suspicion that the virus had come to Europe earlier than it was officially registered. Based on the retrospective testing of respiratory swabs, this group of researchers detected one SARS-CoV-2-positive case in France in late December 2019 (7).

An important conclusion is that VH is a very useful post-mortem sample for SARS-CoV-2 preservation, as it allowed us to detect the virus 3 months after the death, especially in cases when the virus has not been isolated from the blood (8–11). We isolated the virus from VH, although VH underwent freezing at −20°C and subsequent thawing and the confirmatory analysis was done on that thawed sample a few weeks after the first one. This suggests that SARS-CoV-2 is more resistant to temperature changes. Recently published manuscripts showed different results regarding the presence of SARS-CoV-2 in the ocular structures (12–15). In the study of Sawant et al., vitreous swabs were positive for SARS-CoV-2 RNA in 2/10 patients with COVID-19 (14), while one other study did not find SARS-CoV-2 RNA in the vitreous fluid of COVID-19-positive patients (13). On the other hand, Casagrande et al. showed that the prevalence rate of SARS-CoV-2 RNA in the retinal biopsies of patients with COVID-19 is 21% (3/14) (15). Therefore, the possibility of infiltration of VH via retinal vasculature could not be eliminated. VH could be used as a valuable specimen for post-mortem virological analyses, as it stays stable and preserved for a long time. The sequencing of the viral RNA is undoubtedly confirmation that this was in fact SARS-CoV-2, while the possibility of a falsely positive result is excluded.

In the presented case, clinical, radiological, and biochemical findings are in accordance with the severe form of COVID-19. Macroscopically, it is typical of COVID-19 pneumonia to be presented with large and heavy lungs due to retained fluid, as in the presented case. Edler et al. noticed that the lung surface sometimes showed signs of pleurisy and mosaic-like pattern of pale fields and slightly protruding dark purple sections with prominent capillary drawing (16). Also, cut sections of the lungs revealed either ubiquitously dark red or, alternately, faded appearance. In some COVID-19 deaths, purulent respiratory tract infection with abscessed bronchopneumonia was observed (16–18), which is macroscopically different from our findings. Here, the main HP finding was DAD in exudative and early organizing phases, in accordance with the recently published studies (19, 20). Capillary congestion and multifocal microthrombotic disease in capillaries and small vessels were noticed and described in some papers (20). In comparison to other published data, we only detected scattered hyaline microthrombi in the lung capillaries (21, 22). In addition, an increased number of megakaryocytes in the lungs (and other organs) is described in COVID patients, along with some COVID-19-unrelated conditions, like intravascular coagulation, acute infection, shock, and fever (21, 23).

Based on this case, it appears that SARS-CoV-2 has been spreading much earlier than it was considered and that COVID-19 was probably the cause of much-reported pneumonia of unknown origin in early 2020. Important insights into this novel disease and its course can be obtained by post-mortem HP and molecular analyses of the VH, as an excellent sample for SARS-CoV-2 detection, in patients with pneumonia of unknown origin who died in the last one year.

## Data Availability Statement

The datasets generated for this study can be found in online repositories. The names of the repository/repositories and accession number(s) can be found below: https://www.ncbi.nlm.nih.gov/nuccore/MW471658.1/.

## Ethics Statement

Written informed consent was obtained from the individual(s), and minor(s)' legal guardian/next of kin, for the publication of any potentially identifiable images or data included in this article.

## Author Contributions

MB, IS, TA, and AB: drafted manuscript. IS, OS, VP, SS, ZM, and BR: autopsy and pathohistological and molecular analyses. TA, ID, and SD: review of literature. All authors: revised and prepared together the final version of manuscript.

## Conflict of Interest

The authors declare that the research was conducted in the absence of any commercial or financial relationships that could be construed as a potential conflict of interest.

## Footnotes

^1^https://www.who.int/docs/default-source/coronaviruse/real-time-rt-pcr-assays-for-the-detection-of-sars-cov-2-institut-pasteur-paris.pdf (accessed on September 10, 2020).

## Supplementary Material

Supplementary Figure 1 SYBR green RT PCR amplification using Pasteur primers. Linear scale representation. Order of Ct values: 34 for IP2, 33 for IP4, 40 for internal (human) control.

Supplementary Figure 2 SYBR green RT PCR amplification using Pasteur primers. Log scale representation. Order of Ct values: 34 for IP2, 33 for IP4, 40 for internal (human) control.

Supplementary Figure 3 Result of RT-PCR assay.
