# Supplementary figures and images for: Case Report: Post-mortem Histopathological and Molecular Analyses of the Very First Documented COVID-19-Related Death in Europe
Source: Front Med (Lausanne). 2021 Feb 19;8:612758. doi: 10.3389/fmed.2021.612758 (PMC7935505; doi:10.3389/fmed.2021.612758)

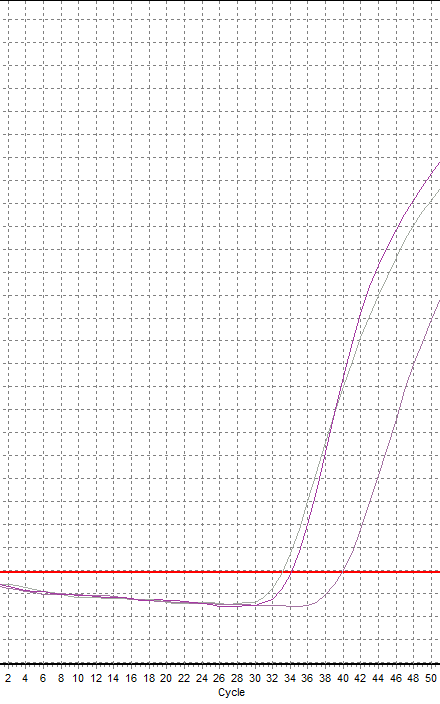

Supplement: Supplementary Figure 1 — SYBR green RT PCR amplification using Pasteur primers. Linear scale representation. Order of Ct values: 34 for IP2, 33 for IP4, 40 for internal (human) control. [file Image_1.TIFF]

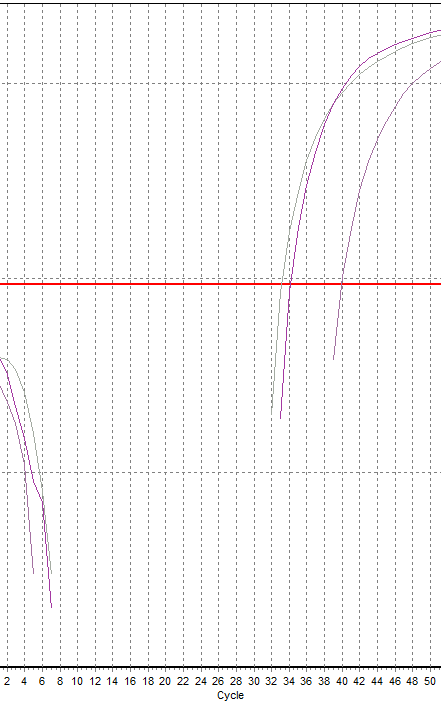

Supplement: Supplementary Figure 2 — SYBR green RT PCR amplification using Pasteur primers. Log scale representation. Order of Ct values: 34 for IP2, 33 for IP4, 40 for internal (human) control. [file Image_2.TIFF]

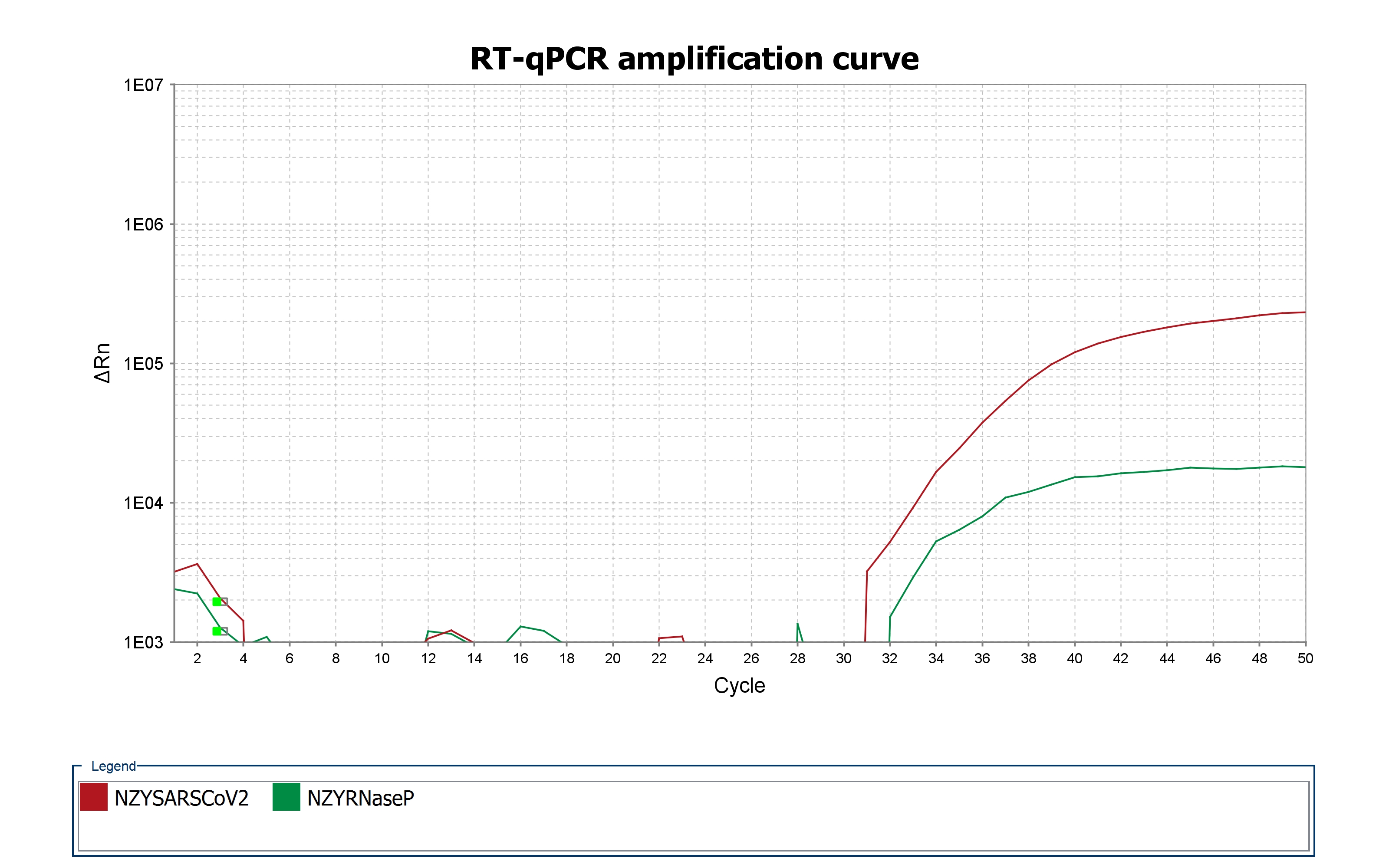

Supplement: Supplementary Figure 3 — Result of RT-PCR assay. [file Image_3.JPEG]
